# Membrane Potential-requiring Succinate Dehydrogenase Constitutes the Key to Propionate Oxidation and Is Unique to Syntrophic Propionate-oxidizing Bacteria

**DOI:** 10.1264/jsme2.ME22111

**Published:** 2023-04-19

**Authors:** Tomoyuki Kosaka, Yuka Tsushima, Yusuke Shiota, Takayuki Ishiguchi, Kazuo Matsushita, Minenosuke Matsutani, Mamoru Yamada

**Affiliations:** 1 Research Center for Thermotolerant Microbial Resources, Yamaguchi University, Yamaguchi 753–8315, Japan; 2 Graduate School of Science and Technology for Innovation, Yamaguchi University, Yamaguchi 753–8315, Japan; 3 Graduate School of Agriculture, Yamaguchi University, Yamaguchi 753–8315, Japan; 4 Faculty of Agriculture, Yamaguchi University, Yamaguchi 753–8315, Japan; 5 NODAI Genome Research Center, Tokyo University of Agriculture, Setagaya-Ku, Japan

**Keywords:** propionate oxidation, hydrogen production, succinate dehydrogenase, membrane potential

## Abstract

Propionate oxidation in *Pelotomaculum thermopropionicum* is performed under a thermodynamic limit. The most energetically unfavorable reaction in the propionate oxidation pathway is succinate oxidation. Based on previous genomic and transcriptomic ana­lyses, succinate oxidation in *P. thermopropionicum* under propionate-oxidizing conditions is conducted by the membrane-bound forms of two succinate dehydrogenases (SDHs). We herein examined the activity of SDH, the mechanisms underlying the succinate oxidation reaction in *P. thermopropionicum*, and the importance of the protein sequences of related genes. SDH activity was highly localized to the membrane fraction. An ana­lysis of the soluble fraction revealed that fumarate reductase received electrons from NADH, suggesting the involvement of membrane-bound SDH in propionate oxidation. We utilized an uncoupler and inhibitors of adenosine triphosphate (ATP) synthase and membrane-bound SDH to investigate whether the membrane potential of *P. thermopropionicum* supports propionate oxidation alongside hydrogen production. These chemicals inhibited hydrogen production, indicating that membrane-bound SDH requires a membrane potential for succinate oxidation, and this membrane potential is maintained by ATP synthase. In addition, the phylogenetic distribution of the flavin adenine dinucleotide‐binding subunit and conserved amino acid sequences of the cytochrome *b* subunit of SDHs in propionate-oxidizing bacteria suggests that membrane-bound SDHs possess specific conserved amino acid residues that are strongly associated with efficient succinate oxidation in syntrophic propionate-oxidizing bacteria.

Propionate oxidation, performed by microorganisms in various environments, is energetically unfavorable, particularly in the absence of electron acceptors; the standard Gibbs free energy change is positive for the oxidation reaction (Schink, 1997). Therefore, propionate oxidation is an unfavorable reaction in methanogenic environments (Schink, 1997; Leng *et al.*, 2018). Propionate-oxidizing bacteria reportedly have a syntrophic relationship with hydrogenotrophic methanogens because hydrogen production due to propionate oxidation is facilitated by the consumption of hydrogen by methanogens (Leng *et al.*, 2018). Although hydrogenotrophic methanogens enhance propionate metabolism in propionate-oxidizing bacteria, bacteria may perform propionate oxidation close to the thermodynamic equilibrium (Jackson and McInerney, 2002). These bacteria possess a specific metabolic mechanism related to the oxidation pathways of substrates, particularly propionate.

Two metabolic pathways of propionate oxidation in syntrophic propionate-oxidizing bacteria have been identified: the methylmalonyl coenzyme A (MMC) pathway (Houwen *et al.*, 1990; Kosaka *et al.*, 2006) (Fig. S1A) and *Smithella* pathway (de Bok *et al.*, 2001). The MMC pathway is utilized by most isolated propionate-oxidizing bacteria (Leng *et al.*, 2018), apart from *Smithella* species (Dyksma and Gallert, 2019). It converts propionate to acetate and carbon dioxide and involves 10 reactions and 3 substrate oxidation steps: malate, pyruvate, and succinate oxidation. Under standard conditions, these oxidation reactions are thermodynamically unfavorable, with succinate oxidation being the most unfavorable (Stams and Plugge, 2009). In addition, in the MMC pathway, membrane-associated protein complexes may be solely responsible for succinate oxidation (Müller *et al.*, 2010). Using menaquinone as an electron acceptor, membrane-bound succinate dehydrogenase (SDH) catalyzes succinate oxidation in the MMC pathway (Schink, 1997; Stams and Plugge, 2009; Leng *et al.*, 2018). The membrane potential maintained by adenosine triphosphate (ATP) synthase, also known as reverse electron transport, potentially facilitates the SDH-induced reduction of menaquinone. This succinate oxidation-requiring membrane potential has been proposed in *Syntrophobacter wolinii*, a mesophilic propionate-oxidizing bacterium (Schink, 1997). However, the succinate oxidation reaction has not yet been investigated in thermophilic propionate-oxidizing bacteria. *Pelotomaculum thermopropionicum* SI grows optimally at 55°C (Imachi *et al.*, 2002) and was isolated in a thermophilic upflow anaerobic sludge blanket reactor from granular sludge (Imachi *et al.*, 2000). A genomic ana­lysis revealed that *P. thermopropionicum* has two types of SDHs (Kosaka *et al.*, 2006, 2008) (Fig. S1B). One SDH is membrane bound (SDH1), while the other is cytoplasmic SDH (SDH2), which has not yet been examined in detail. A transcriptome ana­lysis revealed that SDH1 was highly expressed when propionate was used as a substrate and cocultured with a methanogen (Kato *et al.*, 2009). Nevertheless, these hypotheses are solely based on genome sequences and transcriptomic data, and the existence of these enzymes has yet to be confirmed. Furthermore, to the best of our knowledge, the relationship between hydrogen production and the membrane potential remains unclear. We herein biochemically analyzed SDH activity in *P. thermopropionicum* cells. We also examined hydrogen production from propionate in a *P. thermopropionicum* monoculture in the presence of several inhibitors. In addition, we performed a sequence homology-based ana­lysis of the catalytic domains of SDH1 and SDH2 to elucidate the genetic background and phylogenetic differences between proteins and to identify their key amino acid residues.

## Materials and Methods

### Hydrogen production, bacterial strain, and growth and incubation conditions

*P. thermopropionicum* SI (DSM 13744) was routinely grown on 18‍ ‍mM fumarate or pyruvate in 50‍ ‍mL WY medium at 55°C in a 120-mL serum vial with a butyl rubber seal. WY medium containing 0.01% yeast extract in W medium was prepared as previously described (Kosaka *et al.*, 2014). In the enzyme assay, cells were precultured in 50‍ ‍mL WY medium containing 18‍ ‍mM fumarate as the substrate. All precultured cells were directly inoculated into 6 L WY medium containing 18‍ ‍mM fumarate and propionate as substrates in a 10-L medium bottle filled with N_2_:CO_2_=80:20 gas and sealed with a butyl rubber and plastic cap. The culture was incubated under static conditions at 55°C. Regarding propionate hydrogen production, cells were cultured in 50‍ ‍mL WY medium containing 18‍ ‍mM pyruvate as a substrate. The preculture was typically performed for 2‍ ‍d and then inoculated when growth reached the stationary phase (optical density at 600‍ ‍nm [OD_600_] of approximately 0.25–0.3). Regarding the direct inoculation, the preculture (5‍ ‍mL) was directly inoculated into new media. In the wash inoculation, the preculture was washed as follows: cells were collected from 50‍ ‍mL of the preculture via centrifugation. Cells were then suspended in 1‍ ‍mL WY medium after being washed with fresh WY medium three times. One hundred microliters of the cell suspension was inoculated into fresh 50‍ ‍mL WY medium with 18‍ ‍mM propionate in a butyl rubber-sealed vial. The vial was incubated at 55°C for ~40‍ ‍d, and hydrogen was periodically measured in the headspace. When a constant hydrogen production rate was observed, the headspace was substituted with N_2_:CO_2_=80:20 gas for 3‍ ‍min in a process known as gas exchange to remove existing hydrogen and reduce oxygen contamination, and the vial was then incubated at 55°C. One hundred microliters of each chemical reagent solution was added via a syringe (Terumo). Carbonyl cyanide m-chlorophenylhydrazone (CCCP), 2-thenoyltrifluoroacetone (TTFA), and N,N-dicyclohexylcarbodiimide (DCCD) were dissolved in pyridine. The additive cofactors were dissolved in water and sterilized by filtration.

### Preparation of membrane, soluble, and dialyzed soluble fractions

After an incubation at 55°C for several d when the OD_600_ value was >0.15, cells were collected via centrifugation at 5,000×*g* at 4°C for 10‍ ‍min during the stationary phase. The cell pellet was centrifuged again after being washed with saline containing 8.5‍ ‍mg L^–1^ NaCl. The pellet was suspended in 10‍ ‍mL of 10‍ ‍mM potassium phosphate buffer (KPB, pH 7.0) and subjected to 16,000 psi of pressure in a French press (American Instrument Company). Debris was removed via centrifugation at 8,000×*g* at 4°C for 15‍ ‍min, and the supernatant was ultracentrifuged at 100,000×*g* at 4°C for 60‍ ‍min using an ultracentrifuge (himac CP80WX, Hitachi). The resulting precipitate was homogenized in 10‍ ‍mL of 10‍ ‍mM KPB and used as the membrane fraction. The collected supernatant was dialyzed against 1 L of 10‍ ‍mM KPB at 4°C every 6 h. The treated solution was used as a soluble fraction.

### Enzyme assays

Protein concentrations were measured using the Pierce BCA protein assay kit according to the manufacturer’s instructions (Thermo Scientific™). Using a spectrophotometer, routine ana­lyses were conducted at room temperature using 3-mL plastic or 1‍-‍mL quartz cuvettes (UV-1850; Shimadzu). Succinate:phenazine methosulfate (PMS)/2,6-dichloroindophenol (DCIP) oxidoreductase activity was measured at 600‍ ‍nm, 14.52‍ ‍mM^–1^ cm^–1^ was considered to be the mole­cular extinction coefficient of DCIP (Matsushita *et al.*, 1980), and one unit of activity corresponded to a reduction of 1‍ ‍μmol DCIP min^–1^. The reaction solution contained 16.6‍ ‍mM KPB, 20‍ ‍mM succinate, 200‍ ‍μM PMS, and 100‍ ‍μM DCIP, and the reaction was initiated by the addition of succinate. Succinate:ubiquinone-1 (Q_1_) oxidoreductase activity was measured at 275‍ ‍nm, and we considered 12.25‍ ‍mM^–1^ cm^–1^ to be the mole­cular extinction coefficient of Q_1_ (Redfearn, 1967); one unit corresponded to a reduction of 1‍ ‍μmol Q_1_ min^–1^. The reaction solution contained 45.75‍ ‍mM KPB, 20‍ ‍mM succinate, and 50‍ ‍μM Q_1_, and‍ ‍the reaction was initiated by the addition of succinate. NADH:fumarate oxidoreductase activity was measured at 340‍ ‍nm, and 6.22‍ ‍mM^–1^ cm^–1^ was considered to be the mole­cular extinction coefficient of NADH (Matsushita *et al.*, 1987). One unit was equivalent to the oxidation of 1‍ ‍μmol of NADH min^–1^. The reaction solution contained 20‍ ‍mM KPB, 60‍ ‍mM fumarate, and 5‍ ‍μM NADH, and the reaction was initiated by the addition of fumarate.

### Measurement of the hydrogen content

The gas phase of the cultured vial or bottle was collected using a gas-tight syringe (Hamilton) and applied to a gas chromatography device (GC-8A; Shimadzu) equipped with a thermal conductivity detector (TCD) and a 2×3‍ ‍mm stainless steel column containing Unibeads C (60/80 mesh) (GL Science). The temperature of the injection port and detector was 150°C, and that of the column was 145°C. The TCD was set to a current of 60 mA, and the flow rate of the carrier gas argon was 30‍ ‍mL‍ ‍min^–1^. A calibration curve was produced using standard H_2_ gas (GL Sciences).

### Comparison of sequence data retrieval, phylogenetic tree construction, and gene cluster structures

A total of 1,969 genome sequences were retrieved from the NCBI Reference Sequence (RefSeq) FTP website (ftp.ncbi.nlm.nih.gov/genomes/refseq/). To detect the homologous sequence of SDH/fumarate reductase (FDR), flavoprotein subunit A, we performed a BLASTP search (Altschul *et al.*, 1997) against all of the protein-coding sequences from the 1,969 genomes using the amino acid sequences of three functionally validated protein sequences from *Escherichia coli* BW25113 (SdhA: accession no. AIN31199) and *P. thermopropionicum* SI (Sdh1A: BAF59198 and Sdh2A: BAF59672) as the query. The homologous set was selected by BLASTP based on the criteria of an E-value cut-off of 1e-5 and a minimum aligned sequence length coverage of 70% of a query and hit sequence. All hits from each query were collected, and the merged unique sequence data set was used to build the phylogenetic tree. The input sequence was aligned using MUSCLE 3.8.31 at the amino acid sequence level and used for phylogenetic construction (Edgar, 2004a, 2004b). The MEGAX 10.1.8 package was used to generate a phylogenetic tree to study phylogenetic relationships using the neighbor-joining approach (Tamura *et al.*, 2007; Stecher *et al.*, 2020).

To elucidate the structure of the SDH/FDR gene cluster, 10 genes encoded in the region surrounding each hit were collected. Five of these genes were each encoded in the upstream and downstream regions. Therefore, each hit along with 10 surrounding genes were defined as candidates for the structure of the gene cluster. A homologous group of these candidate proteins was constructed by comparing the all-against-all protein sequences of 1,146 hits and their surrounding proteins using BLASTP (Altschul *et al.*, 1997), followed by Markov clustering with an inflation factor of 1.2 (van Dongen and Abreu-Goodger, 2012). By using an E-value cut-off of 1e-5 and a minimum aligned sequence length coverage of 70% of a query and hit sequence, BLASTP identified the homologous proteins. We investigated flavoprotein subunit A as well as the relationships between the gene cluster structure and phylogenetic location based on the assigned cluster identification of each candidate and their phylogenetic location in SDH/FDR. The domain search was performed using models from the Pfam (https://pfam.xfam.org) and UniProt (https://www.uniprot.org) databases.

## Results

### Existence of SDH activity in *P. thermopropionicum* membrane fractions

According to genomic data, *P. thermopropionicum* SI possesses two types of SDHs, designated as SDH1 and SDH2 (Kosaka *et al.*, 2006) (Fig. S1C). SDH1 and SDH2 were located on the membrane and in the cytoplasm, respectively. This was proposed because SDH1 had a transmembrane SdhC subunit, which contained five transmembrane domains, while SDH2 did not have a similar subunit (Table S1 and Fig. S1B). To confirm the existence of SDHs on the membrane and in the cytoplasm of *P. thermopropionicum*, the enzyme activity of the membrane and soluble fractions of fumarate- and propionate-cultured cells were measured. Both fractions exhibited succinate:PMS/DCIP oxidoreductase activity; however, it was significantly more active in the membrane fraction than in the soluble fraction (Table 1). Furthermore, succinate:Q_1_ oxidoreductase activity levels in both fractions were similar to that of succinate:PMS/DCIP oxidoreductase activity (Table 1). Since the reduction in Q_1_ was considered to be dependent on the cytochrome *b* subunit SdhC of SDH1, which had transmembrane regions (Table S1), succinate oxidation in *P. thermopropionicum* was conducted by SDH1 on the membrane. The soluble fraction exhibited higher NADH:fumarate oxidoreductase activity than the membrane fraction (Table 1), indicating that the reduction of fumarate occurred in the cytoplasm using NADH as an electron donor.

### Conditions of the *P. thermopropionicum* cell preculture for hydrogen production from propionate

**Fig. 1.**
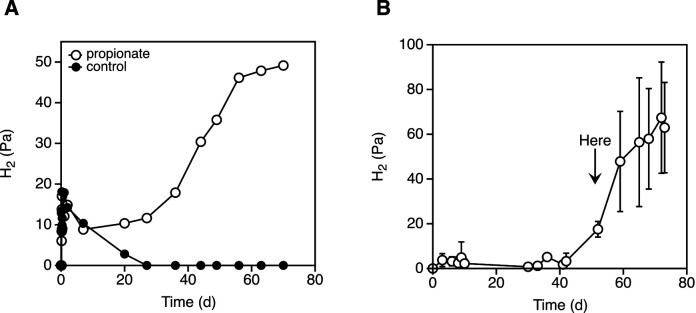
Propionate hydrogen production by *Pelotomaculum thermopropionicum* cells. Hydrogen in the headspace of vials was detected via gas chromatography. (A) Time course of the partial pressure of hydrogen in the headspace of vials with media with (open circles) or without (closed circles) propionate. An aliquot of the preculture with pyruvate-grown cells was directly inoculated. (B) A washing inoculation was used to conduct repeated propionate incubations. Error bars represent the standard deviations for each of the three samples. The arrow indicates the typical timing of gas exchange.

**Fig. 2.**
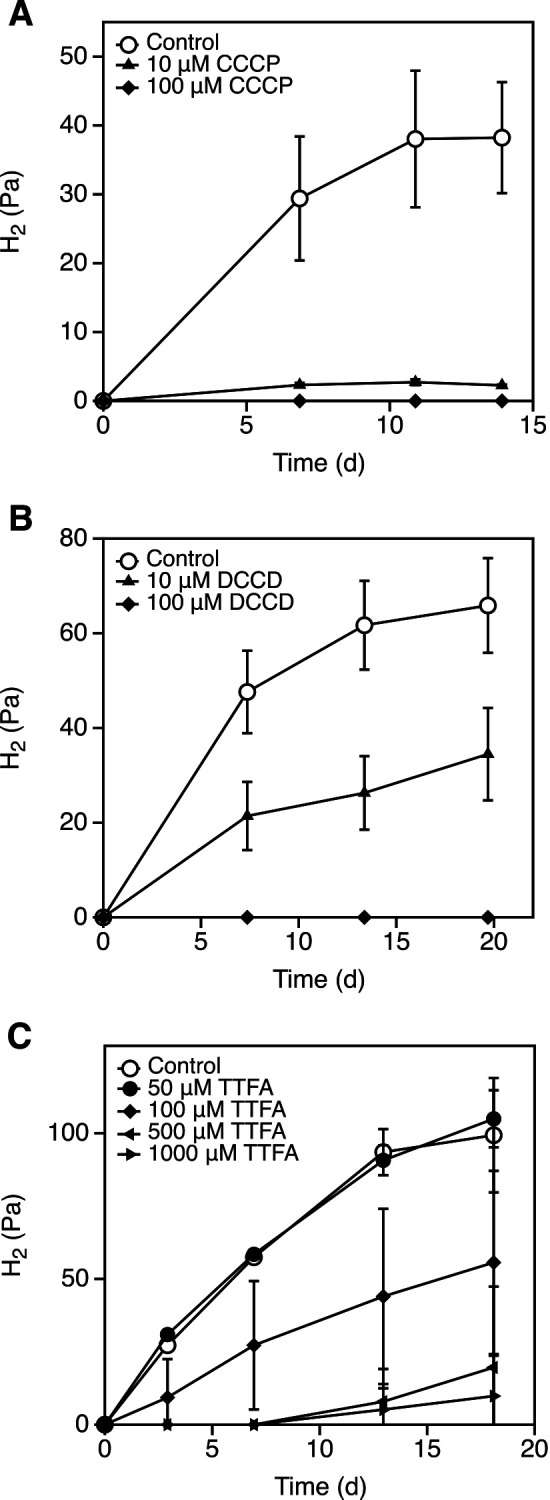
Effects of inhibitors on propionate hydrogen production by *Pelotomaculum thermopropionicum*. Inoculated cells were washed before the propionate incubation. Prior to the addition of chemicals, gas exchange was conducted. At a final concentration of 15.5‍ ‍mM, pyridine was used as an inhibitor solvent and as a control. The inhibitors used were (A) carbonyl cyanide m-chlorophenylhydrazone (CCCP), (B) N,N-dicyclohexylcarbodiimide (DCCD), and (C) 2-thenoyltrifluoroacetone (TTFA). Error bars represent the standard deviations for each of the three samples.

Hydrogen production from propionate has been reported in *P. thermopropionicum* (Kosaka *et al.*, 2014). Since the accumulation of hydrogen inhibits the growth of *P. thermopropionicum* during an incubation with propionate, cell growth does not occur when monocultured in propionate; however, when cell activity is present, a very small amount of hydrogen is produced by cells. However, the incubation period required for hydrogen production was markedly longer, *ca.* 40‍ ‍d, than that reported in a previous study involving *S. wolinii*, a mesophilic propionate-oxidizing bacterium, which produced hydrogen at *ca.* 5‍ ‍h (Schink, 1997). One reason for this difference in the incubation period is the conditions under which *S. wolinii* and other syntrophic, butyrate-oxidizing bacteria were cocultured with methanogens inhibited with bromoethanesulfonate (Wallrabenstein and Schink, 1994; Schink, 1997), whereas *P. thermopropionicum* were monocultured cells (Kosaka *et al.*, 2014). To reduce the time required for propionate hydrogen production, we investigated preculture conditions and culture additives. Propionate hydrogen production via a direct inoculation was observed when cells were inoculated in a preculture for 2–3‍ ‍d with pyruvate as a substrate. The partial pressure of hydrogen was slightly reduced for ~10‍ ‍d, after which it increased to 50 Pa and reached a plateau ~40‍ ‍d later (Fig. 1A). Hydrogen levels did not increase in the absence of propionate (Fig. 1A). Similar results were obtained when washed cells were inoculated; however, initial hydrogen production was reduced (Fig. 1B). When cells were precultured with fumarate, and even when they were inoculated at a high cell density, an increase in hydrogen was not observed for at least 80‍ ‍d (data not shown). The difference in the results obtained among preculture substrates may have been due to enzyme expression because the pyruvate and fumarate cultures produced propionate and succinate, respectively, and the operon-like gene cluster coding the enzymes related to the MMC pathway was not highly expressed in the fumarate culture of *P. thermopropionicum* (Kato *et al.*, 2009). The timing of the inoculation of the preculture did not affect the time required to increase the level of hydrogen; however, the partial pressure of hydrogen observed immediately following the inoculation had changed (data not shown). Furthermore, the addition of 200 nM cofactors, including cobalamin, pantothenate, thiamine, and biotin, into the media with propionate before the cell incubation did not affect the incubation period needed for an increase in the level of hydrogen (data not shown). Although we did not identify any conditions to shorten the period of hydrogen production, we noted high reproducibility when the preculture was performed using pyruvate as a substrate and cells were incubated for >40‍ ‍d (Fig. 1B). Following gas exchange in the headspace of the vial producing hydrogen from propionate, the partial pressure of hydrogen had finally reached 40–100 Pa (Fig. 2).

### Effects of an uncoupler and inhibitors on hydrogen production from *P. thermopropionicum* incubated in propionate-containing media

Under propionate-oxidizing conditions, succinate oxidation constituted the first oxidation step in the MMC pathway (Fig. S1A). This oxidation reaction generated menaquinol, which is required for hydrogen production, and there were no other predicted enzymes besides SDH that produced menaquinol under propionate-oxidizing conditions (Fig. S1B). Furthermore, succinate oxidation was largely responsible for hydrogen production from propionate in *P. thermopropionicum*. Therefore, membrane-bound SDH appeared to be the key enzyme in the MMC pathway. In addition, succinate oxidation in a mesophilic propionate-oxidizing bacterium was previously shown to be dependent on the membrane potential maintained by ATP synthase (Schink, 1997; Stams and Plugge, 2009). To clarify whether succinate oxidation in *P. thermopropionicum* depended on the membrane potential, we examined the inhibitory effects of the uncoupler CCCP on hydrogen production in *P. thermopropionicum* cells incubated with propionate. The addition of 10‍ ‍μM CCCP inhibited hydrogen production from propionate, while 100‍ ‍μM CCCP completely suppressed hydrogen production (Fig. 2A). Furthermore, we measured propionate hydrogen production using DCCD, an ATP synthase inhibitor. Propionate hydrogen production was reduced by 10‍ ‍μM DCCD and completely inhibited by 100‍ ‍μM DCCD (Fig. 2B). These results indicate that *P. thermopropionicum* requires an ATP synthase-maintained membrane potential for propionate hydrogen production. To clarify the relationship between quinones and propionate hydrogen production, we utilized TTFA, which competitively inhibits quinone-binding sites (Grivennikova and Vinogradov, 1982; Horsefield *et al.*, 2006). We observed a decrease in hydrogen production following the addition of >100‍ ‍μM TTFA (Fig. 2C). TTFA also inhibited succinate:Q_1_ oxidoreductase activity in the membrane fraction to a small degree (Fig. S3). These results suggest that membrane-bound SDH1 was essential for succinate oxidation during hydrogen production by *P. thermopropionicum* incubated in propionate-containing media.

### Phylogenetic distribution of flavoprotein subunits and importance of the cytochrome b subunit of SDH

**Fig. 3.**
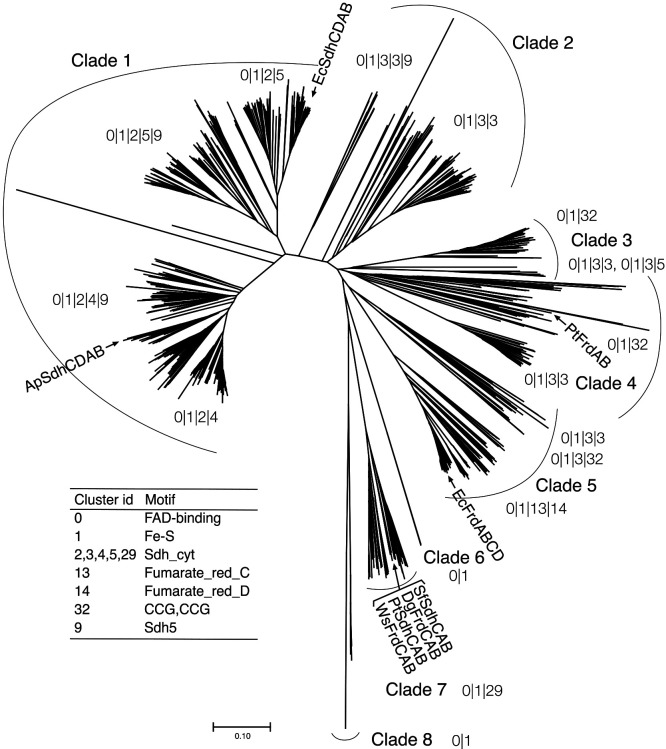
Unrooted neighbor-joining phylogenetic tree of flavoprotein subunits of succinate dehydrogenase/fumarate reductase. A phylogenetic tree of 1,146 homologous protein sequences was constructed using the MEGAX 10.1.8 software package (Tamura *et al.*, 2007; Stecher *et al.*, 2020). The bar on the scale represents 0.1 substitutions per site. Each clade number is listed in Table S3. The numbers, which are cluster IDs, separated by “|” attached to each clade indicate the gene cluster structure in the clade containing each flavoprotein. The inset table displays the cluster ID as well as specific protein motifs listed in Table S4. Gene clusters were found in the strains depicted by the arrows: ApSdhCDAB, *Acetobacter pasturianus*; EcSdhCDAB, EcFrdABCD, *Escherichia coli*; PtFrdAB (SDH2), PtSdhCAB (SDH1), *Pelotomaculum thermopropionicum*; SfSdhCAB, *Syntrophobacter fumaroxidans*; DgFrdCAB, *Desulfovibrio gigas*; and WsFrdCAB, *Wolinella succinogenes*.

**Fig. 4.**
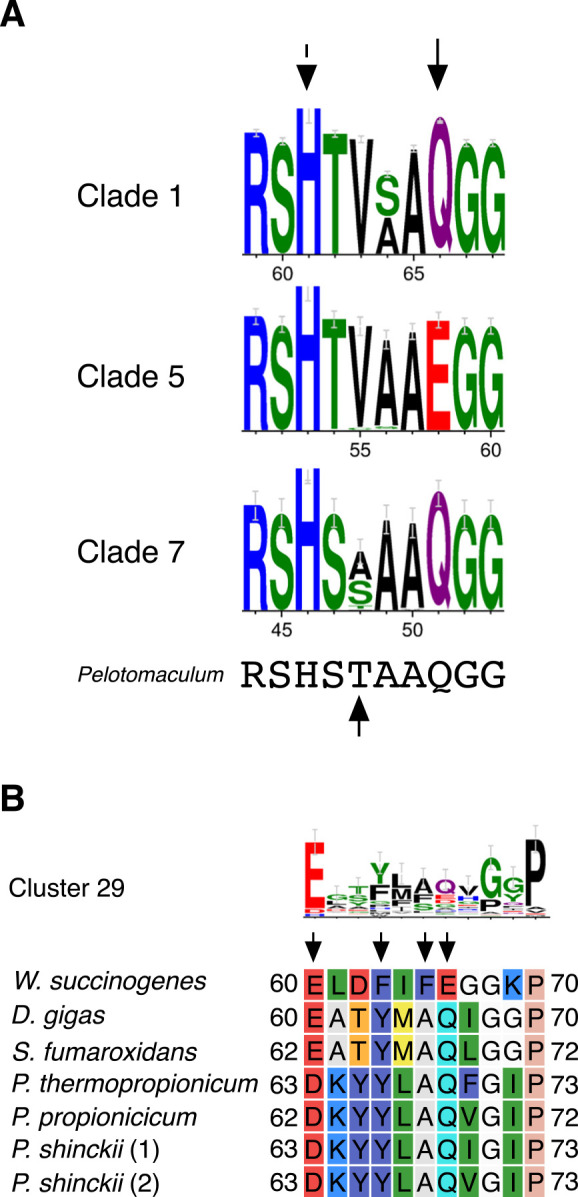
Conserved regions in constructed alignments. The logos depicted are (A) FAD-binding motifs for clades 1, 5, 7, and *Pelotomaculum* or (B) the putative menaquinone-binding site of cluster 29 containing the SdhC subunit. These logos were created using Weblogo (http://weblogo.berkeley.edu) and the alignment dataset shown in Fig. 3 or Fig. S2. The illustrated alignment was partially reconstructed from Fig. S2.

The importance of membrane-associated SDH in *P. thermopropionicum*, a thermophilic propionate-oxidizing bacterium, has increased interest in conserving the amino acid sequence of SDH subunits in propionate-oxidizing bacteria. SDH comprises three or four subunits, including the flavoprotein subunit, SdhA, the Fe-S cluster subunit, SdhB, and the cytochrome *b* subunit, SdhC (with SdhD) (Lancaster, 2013). To examine the phylogenetic distribution of SDH, we compared homologous flavoprotein subunit protein sequences. In the SdhA and FrdA homolog phylogenetic tree, the flavoprotein subunits SDH and FRD,‍ ‍respectively, indicated that the Sdh1A of *P. thermopropionicum* was contained within clade 7 (Fig. 3). Although this phylogenetic ana­lysis was based on protein sequence similarities and did not necessarily provide a phylogenetic classification, clade 7 contained the SdhA of the mesophilic syntrophic propionate-oxidizing bacterium *Syntrophobacter fumaroxidans* (Fig. 3). Conserved amino acid sequences were observed among clade 1, containing *E.‍ ‍coli* SdhA, clade 5, containing *E. coli* FrdA, and clade‍ ‍7.‍ ‍Alignment revealed that FAD-binding motifs (PROSITE:PS00504) were similar and also that the most well-known FAD-binding residue His43 (Cheng *et al.*, 2015; Guan *et al.*, 2018) was highly conserved (Fig. 4A). The eighth amino acid was glutamine (Gln) in clades 1‍ ‍and‍ ‍7 and glutamic acid (Glu) in clade 5 (Fig. 4A). Gln‍ ‍and‍ ‍Glu were consistent with the substrate specificities of succinate:ubiquinone oxidoreductase (SQR) and menaquinol:fumarate oxidoreductase (QFR) as succinate and fumarate, respectively (Maklashina *et al.*, 2006). These results suggest that clade 7 belongs to the SQR type. Furthermore, clades 1 and 5 both had valine at the fifth position, which was unique to *Pelotomaculum* (Fig. 4A).

We investigated the relationships between the Fe-S cluster and cytochrome *b* subunits and the phylogeny of the flavoprotein subunit by summarizing the structures of the cluster of protein homologs comprising SDH/FRD based on‍ ‍the phylogenetic tree of the flavoprotein subunit (Fig.‍ ‍3).‍ ‍Although the Fe-S cluster subunit (cluster 1) was always associated with the flavoprotein subunit (cluster 0,‍ ‍FAD-binding motif), the third and fourth components of‍ ‍each clade were distinct (Fig. 3). Cluster 29, which is affiliated with clade 7, contained the SdhC gene of *P.‍ ‍thermopropionicum* and the cytochrome *b* subunits of *S.‍ ‍fumaroxidans*, *Desulfovibrio gigas*, and *Wolinella succinogenes*. The alignment and conserved sequences of cluster 29 suggested that the His motif for heme binding (His93, His120, His143, and His182 for *W. succinogenes*) was highly conserved (Fig. S2). Additionally, the residues related to the E-pathway for transporting protons outside the membrane into the cytoplasm in the cytochrome *b* subunit of *W. succinogenes* (His44, Glu180) (Lancaster *et al.*, 2005; Lancaster, 2013) were previously reported to be His38, Glu164, and Glu193 in the cytochrome *b* subunit of *D. gigas* (Guan *et al.*, 2018). These residues were conserved in *P. thermopropionicum* (His41, Glu181, and Glu199) and *S. fumaroxidans* (His37, Glu167, and Glu196) (Fig. S2). The E-pathway theoretically reduces the membrane potential of succinate oxidation (Lancaster, 2013). These findings suggest that syntrophic propionate-oxidizing bacteria retain the heme-binding and E-pathway motifs. Notably, Asp63 in SdhC of *P. thermopropionicum* was changed from Glu, which is a putative menaquinone-binding site predicted in‍ ‍*D. gigas* (Guan *et al.*, 2018) (Fig. 4B). Furthermore, the‍ ‍residue was conserved in the SdhCs of the obligate syntrophic propionate-oxidizing bacteria *Pelotomaculum propionicicum* and *Pelotomaculum shinckii* (Hidalgo-Ahumada *et al.*, 2018) (Fig. 4B), suggested that this amino acid residue evolved in syntrophic propionate-oxidizing bacteria requiring the membrane potential for succinate oxidation.

## Discussion

The present results suggest that the SDH of *P. thermopropionicum*, the key oxidizing enzyme directly related to propionate hydrogen production, localizes to the membrane and that the enzyme complex responsible needs to contain the membrane-integrated subunit. A sequence ana­lysis predicted that SDH (SDH1) possesses a quinone pocket in SdhC (Table S1) and transfers electrons to menaquinone, corresponding to membrane-bound Q_1_ reductase activity (Table 1). In addition, we previously demonstrated that the expression levels of the genes encoding SDH1 (PTH_1016-1018) were higher than those of the genes encoding SDH2 (PTH_1492-1490) under conditions of syntrophic propionate oxidation (Kato *et al.*, 2009). These results indicate that SDH1 is primarily an SDH of *P. thermopropionicum*.

Furthermore, the results obtained herein revealed that SDH2 is a cytoplasmic FRD that receives electrons from NADH (Table 1), which is not associated with propionate oxidation. Therefore, we propose that *sdh2A* is *frdA* and *sdh2B* is *frdB*. The electrons required to reduce fumarate by FRD most likely originate from adjacent clustered *PTH_1492* encoding multiple domains containing FrhB, which are the hydrogenase/dehydrogenase beta subunit of coenzyme F420, N terminus (IPR007516), coenzyme F420 hydrogenase/dehydrogenase beta subunit, C terminus (IPR007525), and 4Fe-4S ferredoxin-type iron-sulfur binding domain (IPR017896). However, an additional subunit that oxidizes NADH and transfers electrons to FRD may be required. In the genome of *P. thermopropionicum*, several genes exhibit possible NADH-oxidizing domains, such as NAD_binding_1 (Pfam No. PF00175), oxidized_FMN (PF00724), and Complex1_51K (PF01512). These domains containing genes include PTH_1405 (NAD_binding_1); PTH_0267, PTH_0595, and PTH_0596 (Oxidored_FMN); PTH_2011, PTH_1378, and PTH_2648 (Complex1_51K) (Table S2). The appropriate gene cannot be identified by the presence of a domain; however, the genes in *P. thermopropionicum* may be coupled to FRD. Furthermore, cytoplasmic FDRs are present in the syntrophic propionate-oxidizing bacterium *S. fumaroxidans* (Sfum_4092-4095, Sfum_1998-2000), which lacks heme groups and a predicted membrane-integrated domain cytoplasmic *b*-like (Müller *et al.*, 2010).

The membrane-bound SDH of *P. thermopropionicum* required an ATP synthase-maintained membrane potential for succinate oxidation. This was necessary because propionate hydrogen production by *P. thermopropionicum* required a membrane potential (Fig. 2A), ATP synthase activity (Fig. 2B), and quinones (Fig. 2C). These results are consistent with the predicted reverse electron transport mechanism of membrane-bound SDH from the mesophilic propionate-oxidizing bacterium, *S. wolinii* (Schink, 1997; Stams and Plugge, 2009). TTFA, which affects a broad range of quinone-associated proteins containing a quinone pocket (Grivennikova and Vinogradov, 1982), partially inhibited succinate:Q_1_ oxidoreductase activity (Fig. S3), suggesting that TTFA-causing reductions in SDH hydrogen production warrant further study. One possible TTFA target is the hydrogenase HYD4 because it includes the NrfD subunit, which accepts electrons from the quinone pool (Table S1). Additionally, membrane-bound NiFe-hydrogenase in *E. coli* requires a membrane potential (Pinske *et al.*, 2015). The HYD4 of *P. thermopropionicum* showed significant homology with these genes in *E. coli* (average of 54% positives), indicating that a membrane potential may also be required to drive the HYD4 reaction.

Membrane potential-requiring SDHs have been reported in *Bacillus subtilis* (Schirawski and Unden, 1998) and *Desulfovibrio* species (Zaunmüller *et al.*, 2006). Furthermore, the electrogenic catalysis of SDH has been demonstrated in *Bacillus licheniformis* (Madej *et al.*, 2006). The structure of the subunit and the reaction models of SDHs that utilize the membrane potential for succinate oxidation via transmembrane subunit C (cytochrome *b* subunit) have been proposed in SQR(SDH) of *B. licheniformis* and QFR(FRD) of *W. succinogenes* (Lancaster *et al.*, 2006; Madej *et al.*, 2009; Lancaster, 2013). In the *Wolinella* QFR, a compensatory proton transfer model via the E-pathway present in subunit C contributes an H^+^/e^–^ratio of 0.5 in the quinone-reducing reaction via succinate oxidation, whereas the H^+^/e^–^ratio is 1.0 in subunit C of *B. licheniformis* SQR, which does not utilize the E-pathway (Lancaster, 2013). This difference in ratios in succinate-oxidizing reactions implies the energetic advantage of the E-pathway. *P. thermopropionicum* SdhC conserved several essential amino acid residues of the E-pathway (Fig. S2) and exhibited sufficient homology with the subunits of *Desulfovibrio* (33% identity) and *Wolinella* (27% identity). Subunit C of *D. gigas* QFR has been suggested to utilize the E-pathway in the reversible reaction of quinol oxidation (Guan *et al.*, 2018). These findings suggest that *P. thermopropionicum* SDH utilizes the E-pathway for succinate oxidation. Conversely, the binding of menaquinone to SdhC of *P. thermopropionicum* is crucial for the energetic efficiency of reactions in the SDHs of syntrophic propionate-oxidizing bacteria. Guan *et al.* (2018) proposed Q pockets, menaquinone-binding sites, and related amino acid residues based on the structure of subunit C of *Desulfovibrio* QFR. However, SdhC of *P. thermopropionicum* conserved these amino acid residues for heme binding in subunit C of *D. gigas* QFR (Fig. S2). Other residues associated with menaquinone binding in syntrophic propionate-oxidizing bacteria observed in the alignment (Fig. 4B) may be of greater importance for the menaquinone-specific interaction. These hypotheses require additional biological evidence.

According to the phylogenetic ana­lysis of the flavoprotein subunit SdhA, syntrophic propionate-oxidizing bacteria highly clustered in clade 7 (Fig. 3). Since a correlation was observed between the classification of SdhA and that of the other subunits, SdhB, SdhC, and SdhD (Fig. 3), it is logical to assume that the relationship is significant. This hypothesis has been reported for the respiratory complex protein NADH:ubiquinone oxidoreductase (complex I) (Gabaldón *et al.*, 2005). Additionally, the SdhA subunit is important for substrate specificity and is closely related to FAD binding. The FAD-binding motif in the homologs of SdhA suggests that clustered SdhA in clade 7 is a type of SDH, not FRD (Fig. 4A). These results indicate that the SDHs of syntrophic propionate-oxidizing bacteria have evolved specifically for these microorganisms and that the associated subunits play a crucial role in their function.

Hydrogen production from propionate oxidation in *P. thermopropionicum* requires a membrane potential, which is important for sustaining efficient methane fermentation. In addition, the efficiency of the energetic reaction of succinate oxidation needs to be considered in the structures of SdhA and SdhC, particularly in syntrophic propionate-oxidizing bacteria. The biological mechanisms underlying energetically efficient propionate oxidation by the unique protein complexes of propionate-oxidizing bacteria will be elucidated by the accumulation of additional biological data, including those on actual cell and heterologous expression.

## Citation

Kosaka, T., Tsushima, Y., Shiota, Y., Ishiguchi, T., Matsushita, K., Matsutani, M., and Yamada, M. (2023) Membrane Potential-requiring Succinate Dehydrogenase Constitutes the Key to Propionate Oxidation and Is Unique to Syntrophic Propionate-oxidizing Bacteria. *Microbes Environ ***38**: ME22111.

https://doi.org/10.1264/jsme2.ME22111

## Supplementary Material

Supplementary Material 1

Supplementary Material 2

## Figures and Tables

**Table 1.** Enzyme activities of membrane and soluble fractions prepared from *Pelotomaculum thermopropionicum* cells.

	Membrane fraction*	Soluble fraction*
Succinate:PMS/DCIP oxidoreductase activity (mU mg^–1^)	96.8±68.1	3.7±2.7
Succinate:Q_1_ oxidoreductase activity (mU mg^–1^)	43.9±22.3	2.9±3.6
NADH:fumarate oxidoreductase activity (mU mg^–1^)	5.7±5.3	35.7±17.7

* ± standard deviations (*n*=4)
