# Supplementary material for: Membrane Potential-requiring Succinate Dehydrogenase Constitutes the Key to Propionate Oxidation and Is Unique to Syntrophic Propionate-oxidizing Bacteria
Source: Microbes Environ. 2023 Apr 19;38(2):ME22111. doi: 10.1264/jsme2.ME22111 (PMC10308238; doi:10.1264/jsme2.ME22111)
Supplement: Supplementary file 1 — Supplementary Material 1 [file 38_22111_s1.pdf]

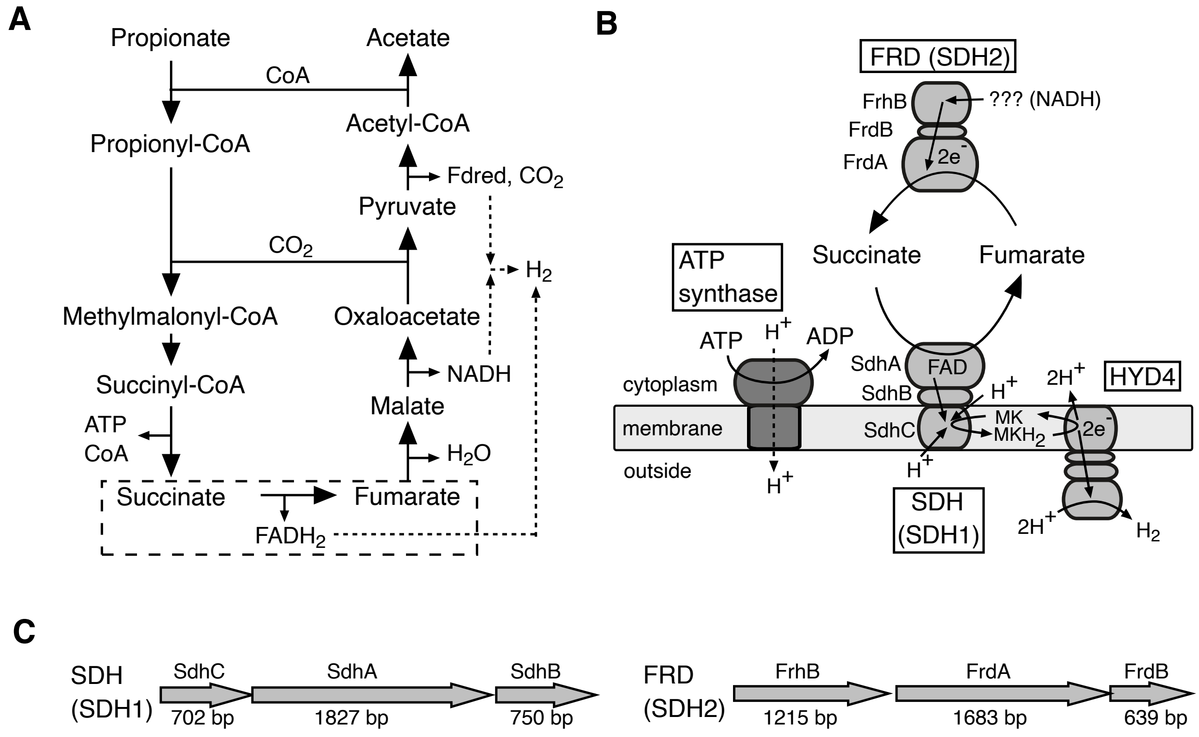

Fig. S1. Schematic diagram of succinate oxidation and hydrogen production in the propionate metabolic pathway of *Pelotomaculum thermopropionicum*

(A) The methylmalonyl CoA pathway, a propionate-oxidizing pathway in *P. thermopropionicum*. Modified from previously published papers [Kosaka, T., Uchiyama, T., Ishii, S., Enoki, M., Imachi, H., Kamagata, Y., Ohashi, A., Harada, H., Ikenaga, H. & Watanabe, K. (2006). Reconstruction and regulation of the central catabolic pathway in the thermophilic propionate-oxidizing syntroph *Pelotomaculum thermopropionicum*. *J Bacteriol* 188, 202–210; Kosaka, T., Kato, S., Shimoyama, T., Ishii, S., Abe, T. & Watanabe, K. (2008). The genome of *Pelotomaculum thermopropionicum* reveals niche-associated evolution in anaerobic microbiota. *Genome Res* 18, 442–448]. Details of the membrane-associated reaction enclosed by the dotted line are in panel (B). (B) Schematic diagram of protein complexes involved in succinate oxidation and fumarate reduction and their relationship to the membrane. The number of subunits of ATP synthase is not exact, but the other complexes are with the predicted number of subunits. (C) Cluster structure within the genome of the genes encoding SDH and FRD. Details on the genes shown in this figure are in Supplemental Table 1. Abbreviations: CoA, coenzyme A; NAD, nicotinamide adenine dinucleotide; FAD, flavin adenine dinucleotide; Fdred, reduced ferredoxin; MK, menaquinone.

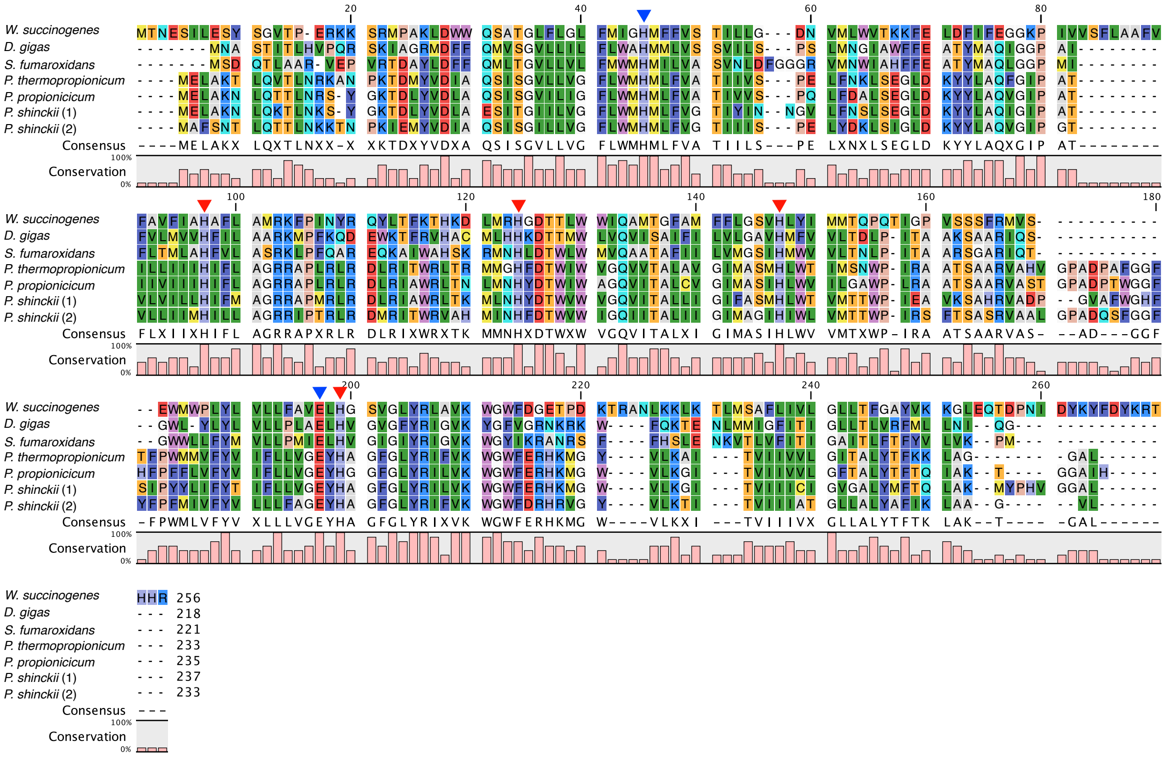

Fig. S2. Multiple alignments of cytochrome *b* subunits of succinate dehydrogenase/fumarate reductase belongs to cluster 29 from several microorganisms involved in clade 7 of the classification of flavoprotein subunit.

The alignment was generated using Clustal Omega 1.2.0 with default parameters in CLC Main Workbench 20.0.4 (http://www. clcbio.com). UniProtKB accession of each sequence of a subunit from a specific strain is as follows: *W. succinogenes*, P17413; *D. gigas*, T2GAT5; *S. fumaroxidans*, *P. thermopropionicum*, A5D3J0; *P. propionicicum*, A0A4Y7RK43; *P. shinckii*, (1)A0A4Y7RCG7 (2)A0A4Y7RAL8. Red arrows indicate conserved histidine residues for heme binding. Blue arrows indicate conserved amino acid residues for E-pathway.

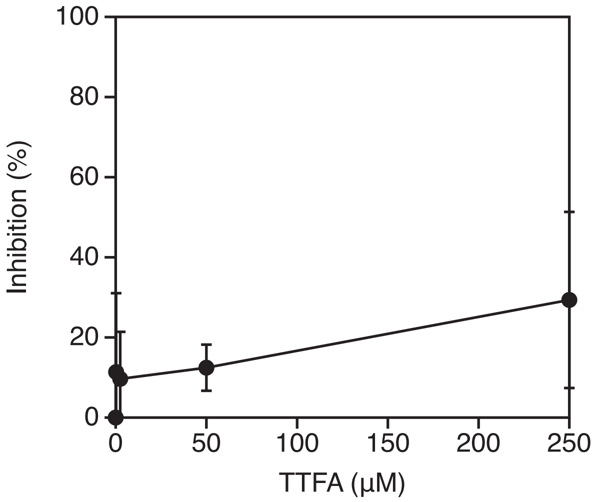

Fig. S3. TTFA inhibition on SDH activity in membrane fractions.

The succinate:Q_1 oxidoreductase activity was measured with the addition of 0.25, 2.5, 50, and 250 μM 2-thenoyltrifluoroacetone (TTFA). TTFA was dissolved in dimethyl sulfoxide and added to the reaction mixture before the addition of succinate to initiate the reaction. Percentage inhibition was calculated on the basis of activity values under 0 μM TTFA conditions. The error bars indicate the standard deviations of the three individual samples.

Table S1. Predicted genes for succinate oxidation and hydrogen production in *P. thermopropionicum*

Enzyme	Locus_tag	UniProt	Protein	Annotation	Electron transfer	Size (aa)	TM *	TAT †
SDH1 SDH	PTH_1016	A5D3J0	Sdh1C > SdhC	succinate dehydrogenase/fumarate reductase, cytochrome b subunit	Succinate <-> MK	233	5	
	PTH_1017	A5D3J1	Sdh1A > SdhA	succinate dehydrogenase/fumarate reductase, flavoprotein subunit		608	0	
	PTH_1018	A5D3J2	Sdh1B > SdhB	succinate dehydrogenase/fumarate reductase, Fe-S protein subunit		249	0	
SDH2 FRD	PTH_1490	A5D270	Sdh2B > FrdB	succinate dehydrogenase/fumarate reductase, Fe-S protein subunit	Fumarate <->?	212	0	
	PTH_1491	A5D271	Sdh2A > FrdA	succinate dehydrogenase/fumarate reductase, flavoprotein subunit		560	0	
	PTH_1492	A5D272	FrhB	coenzyme F420-reducing hydrogenase, beta subunit		404	0	
HYD1	PTH_0668	A5D4I9		Iron only hydrogenase large subunit, C-terminal domain, containing ferredoxin	? <-> H_2	530	0	+
	PTH_0669	A5D4J0	HybA	Fe-S-cluster-containing hydrogenase components 1		271	0	
	PTH_0670	A5D4J1		Hypothetical protein		89	1	
HYD2	PTH_1377	A5D2H3		hypothetical hydrogenase subunit	NADH, Fd red <-> H_2	624	0	
	PTH_1378	A5D2H4	NuoF	NADH:ubiquinone oxidoreductase, NADH-binding (51 kD) subunit		650	0	
	PTH_1379	A5D2H5	NuoE	NADH:ubiquinone oxidoreductase 24 kD subunit		192	0	
HYD3	PTH_2010	A5D0Q2		hypothetical hydrogenase subunit	NADH, Fd red <-> H_2	574	0	
	PTH_2011	A5D0Q3	NuoF	NADH:ubiquinone oxidoreductase, NADH-binding (51 kD) subunit		551	0	
	PTH_2012	A5D0Q4	NuoE	NADH:ubiquinone oxidoreductase 24 kD subunit		162	0	
HYD4	PTH_1701	A5D1L0	HyaA	Ni,Fe-hydrogenase I small subunit	MK <-> H_2	332	0	+
	PTH_1702	A5D1L1	HyaB	Ni,Fe-hydrogenase I large subunit		482	0	
	PTH_1703	A5D1L2	HybA	Fe-S-cluster-containing hydrogenase components 1		277	0	
	PTH_1704	A5D1L3	NrfD	NrfD participates in the transfer of electrons from quinone pool into the terminal components		389	10	

*TM: transmembrane region numbers from UniProt information. †TAT: +, presence of twin-arginine translocation signal peptide.

Table S2. Search of NADH oxidizing domain in *P. thermopropionicum*[a]

Query Pfam domain	Pfam No.	Query Length	Target locus Tag	Target UniProt Accession	Target Length	E-value	Score	Description	Related cluster	Trans-membrane[b]
NAD_binding_1	PF00175	109	PTH_1405	A5D2E4	280	3.1E-12	58.5	2-polyprenylphenol hydroxylase and related flavodoxin oxidoreductases	PTH_1405-1413	0
NAD_binding_1	PF00175	109	PTH_1180	A5D320	287	0.0009	31.3	2-polyprenylphenol hydroxylase and related flavodoxin oxidoreductases	?	0
Oxidored_FMN	PF00724	342	PTH_0267	A5D5M6	641	1.7E-81	285.8	Uncharacterized protein	?	0
Oxidored_FMN	PF00724	342	PTH_0595	A5D4R8	651	1.7E-60	216.8	NADH:flavin oxidoreductases	PTH_0594-0601	0
Oxidored_FMN	PF00724	342	PTH_0596	A5D4R7	649	4.6E-52	189.1	NADH:flavin oxidoreductases	PTH_0594-0601	0
Complex1_51K	PF01512	152	PTH_2011	A5D0Q3	551	5.4E-46	167.8	NADH:ubiquinone oxidoreductase, NADH-binding 51 kD subunit	HYD3 (PTH_2010-2012)	0
Complex1_51K	PF01512	152	PTH_2648	A5CYU7	617	1.9E-44	162.8	NADH:ubiquinone oxidoreductase, NADH-binding 51 kD subunit	PTH_2647-2650	0
Complex1_51K	PF01512	152	PTH_1378	A5D2H4	650	5.6E-43	158.0	NADH:ubiquinone oxidoreductase, NADH-binding 51 kD subunit	HYD2 (PTH_1377-1379)	0

[a]Search was performed by hmmsearch (https://www.ebi.ac.uk/Tools/hmmer/search/hmmsearch) with Pfam HMMs.
[b]Search was performed by DeepTMHMM (https://biolib.com/DTU/DeepTMHMM/)
